# Mental health crisis in Somalia: a review and a way forward

**DOI:** 10.1186/s13033-022-00525-y

**Published:** 2022-02-09

**Authors:** M. Ibrahim, H. Rizwan, M. Afzal, Mamunur Rahman Malik

**Affiliations:** 1 School of Social Work, The University of British Columbia, 2080 Jack Bell Building, Vancouver, V6T 1Z2 Canada; 2 WHO Sudan Country Office, Khartoum, Sudan; 3 Health Research, Monitoring and Evaluation Consultant, Islamabad, Pakistan; 4 WHO Somalia Country Office, Mogadishu, Somalia

**Keywords:** Somalia, Conflict, Humanitarian crisis, Mental health, Community Mental Health

## Abstract

**Background:**

Somalia has been without an effective government since the collapse of the military regime in 1991. Years of conflict, disasters, and insecurity have all contributed to very low scores for most health indicators due to poor governance, protracted conflict, underdevelopment, economic decline, poverty, social and gender inequality, and environmental degradation. The three-decade long protracted conflict has led to widespread psychosocial trauma, social deprivation and substance abuse with devastating consequences on mental health. A WHO study showed Somalia has one of the highest rates of mental illness in the world. The main aim of this study is to assist policy makers in setting priorities for the design and delivery of interventions to promote mental health and psychosocial wellbeing in Somalia.

**Methods:**

The study uses a systematic mapping technique (from January 1991 to May 2020) and data collected from public domain, to collect, collate, and present mental health data mainly from WHO’s Global Health Observatory. Since there is no primary database for Somalia’s public health research, the bibliographic databases used for mental health in this study included Medline, PubMed, CINAHL, PsycINFO, and Google Scholar. Data were extracted using techniques for web data mining for public health.

**Results:**

Systematic mapping of mental health-related issues in Somalia showed that policy-related determinants and mental health services dominated (74.4%), followed by the disaster-related determinants and women’s health consequences (39.3%). The ratio of the number of beds for mental health in general hospitals (per 100,000 population) in Somalia in 2017 is 0.5 compared to the Eastern Mediterranean region (EMR) at 6.4 and globally at 24. One of the biggest casualties of the civil war was loss of essential human resources in healthcare as most either fled the country or were part of the victims of the war.

**Conclusions:**

The vast scale of the mental health problems in Somalia and the priority setting guidelines for interventions to address the issues outlined in this paper, prompt a dire need that the Somali government and its national/international partners should prioritize and emphasize the need to invest in the prevention and the treatment of mental illness across the country.

## Background

Somalia is situated in the northern most region of the Horn of Africa with an estimated population of about 16 million out of which 60% are below the age of 25 years making Somalia one of the youngest nations in the world [1]. The country’s population is generally homogenous in terms of language, culture and religious background, with the overwhelming majority speaking the Somali language. Somalia is politically and administratively a federated country with five regional states namely: Puntland, Jubaland, Galmudug, South West and Hirshabelle. In addition, Somaliland, situated in the north, self-declared independence in 1991 after the collapse of the central government. Somaliland has yet to gain international recognition.

The country has been without an effective national government since the collapse of the military regime in 1991. The disagreement to form a unified government led to inter-militia conflict, which expanded to inter-clan conflict. This unprecedented fall out between the major clan groups claimed thousands of lives, uprooted communities, and displaced millions internally and externally [2]. Alongside the inter-clan conflict, war evolved and attracted different international, regional and local actors including violent militant groups, such as Al-Shabab [2].

Years of conflict, natural disasters, famine and insecurity, all have contributed to very low scores for most humanitarian indicators, suffering from poor governance, protracted internal conflict, underdevelopment, economic decline, poverty, social and gender inequality, and environmental degradation [3, 4]. Despite high mortality caused by civil war and famine, Somalia’s population is growing rapidly due to a high fertility rate (more than six children per woman) and a considerable proportion of people of reproductive age. With each generation being larger than the prior one, Somalia’s population puts a strain on the country’s poor health care and social services [1, 3, 5, 6].

Because about 60% of Somalia’s population is younger than 25 years and were born during the civil war, they do not know what it feels like to live in peace as conflict has been ever-present throughout their lifetime.

Studies of adverse childhood experience have shown that a high prevalence of early childhood trauma causes long-term health effects in adulthood [7].

In Somalia’s recent history, mental health services barely existed as part of the larger government-run health care services [8]. Despite the existence of few colonially built psychiatric units, mental health services were largely outside the purview of the government-run health care services. As such, most Somalis depend on family and community support for mental health and rely on the services of traditional and spiritual healers for treatments [3, 8–10]. One of the biggest casualties of the civil war was loss of essential human resources in health care as most either fled the country or were victims of the war [11]. Hence the country is facing a serious situation of acute and chronic shortage of health professionals. Such a shortage is more profound in the field of mental health where only a handful of professionals provide services across the country [11].

In addition to armed conflict and insecurity, the country has faced climate-related shocks, mainly drought and flooding, displacing about 2.6 million people and exposing them to multiple risks [11]. Currently 5.4 million people—more than one third of the population—are in need of humanitarian assistance [11]. This situation has led to widespread psychosocial trauma, social deprivation and substance abuse, with devastating consequences on mental health. According to a situation analysis by the World Health Organization (WHO) in 2010, Somalia had one of the highest prevalence of mental health problems in the world with one third of Somalis afflicted with some form of psychological disorder [3–6]. However, despite these grim statistics, there is a lack of adequate mental health services in the country with only five low-capacity psychiatric units mostly situated in Somaliland, the relatively stable region [3, 4].

In a country with a fragile health system and ill-equipped formal institutions, affected individuals often seek help from traditional and spiritual healers while others resort to self-medication, substance use or other potentially harmful coping strategies [3, 12]. In the Somali cultural setting, mental health has largely been perceived in binary perspectives of a person being either normal or mad [3]. In this regard, the notion of a continuum of mental health issues ranging from mild, moderate, to severe simply did not exist as part of the cultural and linguistic nosology. Beliefs in the causes of mental illness and treatment in the country are predominately metaphysical and spiritual [3, 4, 9, 12]. Additionally, significant stigma and discrimination in the society conceals mental health issues [3, 13]. Consequently, mental health services are woefully lacking, and human rights abuses are common including chaining or caging at home [3, 13]. WHO estimates that 90% of people with serious mental health problems were chained at least once in their lifetime [3].

Continuous humanitarian crises have had a devastating impact on the Somali civilians. Conflict-related trauma, poverty, unemployment and rampant substance abuse in the country has elicited an explosion of mental health problems [3]. The health care system in Somalia has never developed beyond providing the most basic functions and has negligible capacity to manage monumental challenges from a population health perspective [3, 4].

Mental health and substance use disorders are specifically included in the United Nations Sustainable Development Goals (2015–2030) [14]. The WHO’s Mental Health Strategy and Action Plan (2013–2030) has also set a range of targets aimed at achieving equity through universal health coverage (UHC). Due to the long history of neglect of mental health issues in Somalia, only few partners operate in this area of work. Since 2011, the WHO, in collaboration with the non-governmental organisation (NGO) Gruppo per le Relazioni Transculturali, has worked in mental health [15, 16]. In partnership with Gruppo per le Relazioni Transculturali and Habeeb Mental Health Foundation, the WHO Somali Office launched the Chain-Free Initiative in Somalia. The Initiative aims to increase access to mental health services (facilitating humane treatments in hospitals, at home and in communities), combat discrimination and decrease the number of patients living in chains [16].

Research has shown that improving mental health of the population guarantees wellbeing and social, humane and economic capital of a nation, as one study showed in some middle income countries such as India, Chile and South Africa [17, 18]. The mental health crisis in Somalia requires that government, policy makers and practitioners comprehend the magnitude of the factors that moderate them.

The main aim of this study is to assist mental health professionals, policy makers, government and humanitarian actors in setting priorities for the design and delivery of interventions to promote mental health and psychosocial wellbeing in Somalia.

The study’s objectives are:

- to review existing literature (peer reviewed, grey literature and reports) of mental health in Somalia using a systematic mapping technique;
- to assess the mental health system and other mental health determinants in the context of Somalia;
- to examine, from the existing literature, the impact of the long-standing conflict on the mental wellbeing of Somalis;
- and finally, to propose a framework for priority setting for mental health in Somalia that can translate to other conflict-affected settings.

## Methods

The study is based on a literature search and retrieved (from January 1991 to May 2020) and organised using a systematic mapping technique. Data was collected from the public domain, primarily mental health data from WHO’s Global Health Observatory [19].

To explore a priority setting process for mental health, a systematic mapping approach [20–22] was undertaken by mapping of mental health publications on Somalia from 1991 to date (May 2020). Systematic mapping provides a comprehensive overview of any broad research field such as mental health, to collate, describe and catalogue available evidence relating to a topic or question of interest, as well as providing guidelines and direction for needed actions [21–23]. Thus, mapping results, though not meant to answer a specific question, can be used to identify evidence for policy-relevant questions, knowledge gaps, and provide an information resource for a range of target audiences including, researchers, clinicians and policy makers.

### Systematic mapping process

Systematic mapping was first used in social sciences to reliably register evidence on a subject of interest [21]. At present, however, the definition of ‘systematic mapping’ varies widely in different disciplines. For example, in public health studies the use of systematic mapping has overlapped with ‘scoping review’, while in other health studies the latter term has been considered an important step of the systematic mapping process [21, 22].

The mapping methodology comprised three stages (Table 1). The first two stages involved the systematic and scoping review processes: identification of the mental health domains/issues in Somalia followed by characterisation of the smaller sub-fields used to address the focused areas as described below in Sect. 2.2. The third stage was systematic mapping that included data extraction and mapping of relevant mental health publications. In order to investigate which types of research have been reported in the literature, to identify the areas of study and to identify publication trends over the study period, the mapping process was guided by the following questions:

- What are the research/study types of mental health publications concerning mental health in Somalia?
- What are the main topics addressed in the literature?
- How has the frequency of the mental health studies changed over time?

**Table 1:** Systematic mapping process

	Establishing the systematic mapping team
	Setting the scope and mapping questions to guide the process
Stage 1	Setting inclusion criteria for studies
	Conducting scoping review to confirm the relevance of inclusion criteria and potential questions
	Protocol development
	Searching for evidence
Stage 2	Screening evidence
	Classification scheme: key wording using abstracts (identifying items of analytic interest)
Stage 3	Systematic mapping: information/data extraction and mapping
	Describing and visualising the findings

### Databases and search strategies

The bibliographic databases used to search for mental health-related areas in this study included Medline, PubMed, CINAHL, PsycINFO, Google Scholar, and Social Services Abstracts [21, 22].

In addition to the database search, a web-based search was undertaken to retrieve literature relevant to the Somali mental health situation. Assessment and evaluation reports by NGOs, and agencies of the UN were also consulted to find important mental health information. Moreover, reference lists of key papers, books and articles relevant to Somali mental health were manually searched [24].

The search strategies included combinations of the following terms grouped into broader fields/sub-fields to capture mental health issues in Somalia.

General mental health terms: Somal*, psychiatr*, mental health, mental illness, mental problems, mental disorders and psychosocial.

### Terms addressing mental health determinants:

- Policy-related determinants: policy, legislation, services, human resources, financing and priorities.
- Disaster-related determinants: war trauma, adolescent refugees, forced migrants, urban mental health, women’s health consequences, physical and mental symptoms, and collective trauma.
- Social and economic determinants: gender, stigma, traditional practices and healings, substance use, unemployment and poverty.

### Data extraction and synthesis

Data were extracted using techniques for web data mining for public health [25]. A data extraction tool was employed to capture the mental health indicators identified through search and the extracted data were manipulated into a generic format [26]. To draw reliable conclusions from the assembled body of evidence, synthesis was conducted considering the value of evidence, consistency of any detected effects across studies and investigation of possible reasons for any contradictory observations.

## Results

### Systematic mapping studies addressing mental health-related domains/issues in Somalia

The systematic mapping conducted revealed a wide array of topics across several major research/study areas in the existing literature addressing mental health issues of Somalia.

**Fig. 1:**
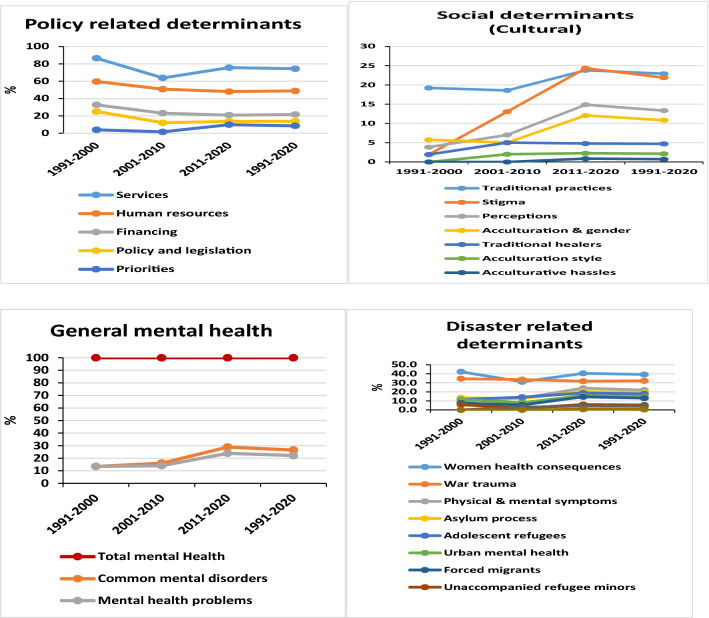
Growth trend of the distribution of peer review publications pertaining to different mental health-related fields and sub-fields, presented as percentage of total peer reviewed publications addressing mental health in Somalia from 1991 to May 2020

All databases searched provided 25,700 records. After exclusion, the number of identified records was reduced to 9340 and 1401 of these could be categorised into one of the domains of interest addressing mental issues in Somalia. The distribution of publications by mental health fields/sub-fields is diagrammatically represented in Fig. 1. The percentages are based on a total of 1401 records.

Among the major searched domains, policy-related determinants and mental health services dominated (1042 records, 74.4%), followed by the disaster-related determinant and women’s health consequences (550 records, 39.3%). A total of 419 (29.9%) records dealt with longitudinal studies, while 361 (28.5%) and 321 (22.9%) records addressed training for mental health professionals and traditional practices, respectively. Among the policy-related domains, services, human resources, financing, policy and legislation, and mental health priorities, were represented with 74.4%, 48.8%, 21.6%, 13.8% and 8.4% records, respectively.

The disaster-related determinants were by far the largest set, comprising eight areas: women’s health consequences (39.3%), war trauma (32.2%), physical and mental symptoms (22%), adolescent refugees (17.8%), forced migrants (13%), unaccompanied refugee minors (5.4%) and collective trauma (0.6%). The disaster-related determinants were followed by social determinants: traditional practices (22.9%), stigma (21.9%), perceptions (13.3%) and traditional healers (4.7%). The behavioural determinants were as follows: self-harm (18.8%), substance abuse (16.1%) and suicide (9.1%).

### Health system determinants of mental health

#### Mental health services

**Fig. 2:**
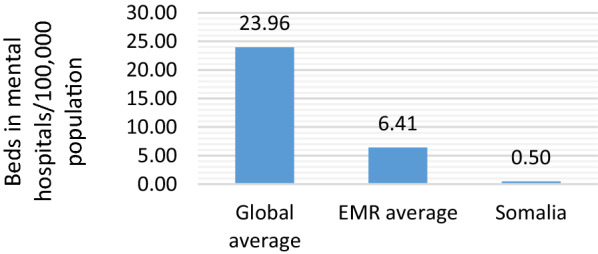
Number of mental hospitals and mental health outpatient facilities per 100,000 population in Somalia in 2016 ( Source: WHO’s Global Health Observatory [19])

Although the number of psychiatric beds has increased slightly in recent past based on the data from 1991 to 2016, the ratio of number of beds for mental health in general hospitals falls abysmally behind the Eastern Mediterranean region (EMR) and the global average (Fig. 2). Therefore, Somalia remains severely underserved in terms of mental health services. Furthermore, community mental health, whether as part of integrated primary health care (PHC) services or stand-alone services, is virtually non-existent across the country [19].

Since 2009, the self-declared independent Somaliland expanded its mental health services across five main cities: Hargeisa, Berbera, Borama, Gabiley and Burao. In total the bed capacity increased to 250 for a population of 4 million [4, 27]. Whereas across the rest of the country, psychiatric units within general hospitals have been established in Puntland (Bosaso) and South Central (Mogadishu, Baidoa and Beledweyne) but the bed ratio remains one of the lowest globally. The rural and remote communities find it more difficult to access and maintain contact with the existing mental health services.

#### Human resources

Although a reliable source of data on human resources for health is lacking in Somalia, owing to dismal conditions of overall human resources for health, there is apparently a severe paucity of mental health workforce in the country [10]. Research shows that the quality of mental health care in low‐ and middle‐income countries (LMIC) faces at least four key challenges: limited resources, weak health system, lack of standardised services, and diverse cultural environments [28]. In this light, limited resources in Somalia are evident with the very low median number of mental health workers.

One study provides evidence that there were only three psychiatrists working in public facilities in Somalia in 2010 [3]. In most regions, only general practitioners acting as psychiatrists are available. In recent years, nurses and social workers have been trained by international NGOs and/or WHO in two short courses of training on mental health conducted initially in Bosaso and later in Hargeisa. Further, the country is currently experiencing a proliferation of for-profit academic institutions offering various academic programmes including health-related ones. However, there is a lack of frameworks and regulation for ensuring quality education at both federal and member state levels. Moreover, want of an accreditation system raises some serious doubts on the basic training and standards for medical professionals engaged in provision of mental health services in Somalia. There is an overall lack of data regarding professional training and continuing professional development [3, 4, 9].

#### Mental health policy and legislation

Somalia’s first ever mental health policy and strategy was formulated in 1986 by the Ministry of Health with the help of WHO. However, due to the beginning of unrest in the late 1980s which eventually led to the collapse of the national government in 1991, the mental health initiatives were never realised [8].

Currently, there is no mental health legislation in place in Somalia to protect the lives, rights and integrity of people with mental illness [4, 9]. Somaliland published a mental health policy in 2012 to set out plans to develop and organise mental health services, including the development of community-based services, training, research and legislation. However, due lack of funding and political will, the policy remains only on paper and is yet to be implemented [4]. Recently the Federal Government of Somalia has moved to redevelop key policies and strategies including mental health and has established a mental health portfolio.

A legal and policy framework is crucial in the mental health sector considering the widespread level of stigma, discrimination and human rights abuse (Human Rights Watch, 2015). The Human Rights Watch 2015 report depicted significant and ongoing human rights violations of persons with severe mental illness in Somaliland [13]. The abuses included, among others, physical violence and chaining or locking up of individuals with mental illness in psychiatric facilities, jails and spiritual healers’ centres or even in families’ backyards. It is estimated that 170,000 people with mental health problems in Somalia are kept in chains. Neglect, inadequate nutrition and poor hygiene is rampant [13, 29].

As the country slowly rises from the ashes of years of anarchy, it is prudent to establish comprehensive and progressive legislation and policies for mental health and other vulnerable sectors in order to serve and protect their rights as equal citizens.

#### Mental health financing

Evidence shows that material, financial and human resources for mental health in most low- and middle-income countries is dismal. In fact, more than 70% of African and 50% of Southeast Asian countries spend less than 1% of their health budget on mental health [30]. In the case of Somalia, the long-standing instability has severely curtailed the government’s ability to provide and fund essential services.

To overcome Somalia’s chronic emergency, the international community has allocated funds to different sectors including health. Except for a comprehensive review published by the World Bank (WB) in 2008 [31], the information of health sector aid financing in Somalia is dismally negligible. In the last decade, horizontal health system strengthening programmes lagged behind vertical programmes. However, since 2010, a more determined health systems strengthening approach has been adopted with integration funding across Somaliland, Puntland and South Central [31–33].

Despite the significant need for mental health services, resource allocation is largely donor-related but even then, very few NGOs and international organisations focus on mental health in Somalia. As such, any meaningful improvement of the mental health system in Somalia is likely to be in jeopardy without significant investment in this sector.

At national and regional levels, no government agencies exist that are mandated to source, inventory, purchase and distribute essential medicines. At the service provision level, essential psychotropic drugs that are crucial for medical treatment and for managing the most acute cases initially are not always available at the end-user level.

### Disaster and trauma-related determinants

Limited epidemiological studies have been conducted determining the prevalence of mental illness among Somalis and Somali refugees. Available research has suggested that refugees are at risk for the development of a variety of psychological disturbances including depression, anxiety and post-traumatic stress disorder (PTSD) [3, 27, 34]. However, inconsistency is found in the results reported about prevalence and severity of mental illness among Somali refugees. The conflicting results in the existing literature may be attributed to the complexity of cross-cultural survey techniques and methodological inconsistency as well as interpersonal variability among patients regarding psychological resiliency and psychopathological symptoms [35]. There are limited studies within the country although two earlier studies show high rates of mental illness in the country with one in three and one in every two households in Somaliland [29] suffering from some form of mental illness.

Recently, the Joint Multi cluster Needs Assessment survey highlighted a substantial prevalence of mental health issues among the affected population in Somalia [10, 34]. Some case studies of individuals from Somalia show high rates of schizophrenia, bipolar disorder, PTSD, depression, mania and psychosis. There are also individual reports of repressed traumatic memories manifested through bouts of depression, anxiety, PTSD, and thoughts of suicide and self-harm.

#### Suicide and self-harm

**Fig. 3:**
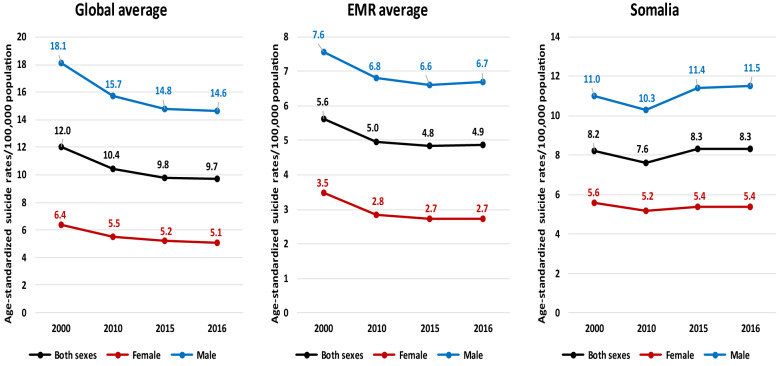
Age-standardised suicide rates per 100,000 population in Somalia from 2000 onwards as compared with as compared with global and EMR averages in the same years ( Source: WHO’s Global Health Observatory [19].)

Available data on age-standardised suicide rates per 100,000 population in Somalia from 2000 onwards are summarised in Fig. 3 and compared with the global and EMR averages for the same years. Although mental health disorders have been reported as one of the highest and affect one third of the Somali population, [3, 15, 36] the suicide rate in the country has been less than the global average. However, the rate in Somalia has been higher than the average suicide rate for the EMR. Figure 3 depicts the number of suicides in Somalia annually from 2000 onwards. It is also evident that the suicide rate has increased in males and females in Somalia from 2000 onwards while the average rate declined globally and in the EMR during this period (Fig. 3).

However, unlike suicide rates, the disease burden of self-harm in Somalia has a declining trend in both sexes since 2000 [37, 38]. Like suicide, self-harm has always been higher in males in Somalia [38]. Contrary to suicides, the disease burden of self-harm has been far lower in Somalia than that in most of the EMR states.

#### Substance use

**Fig. 4:**
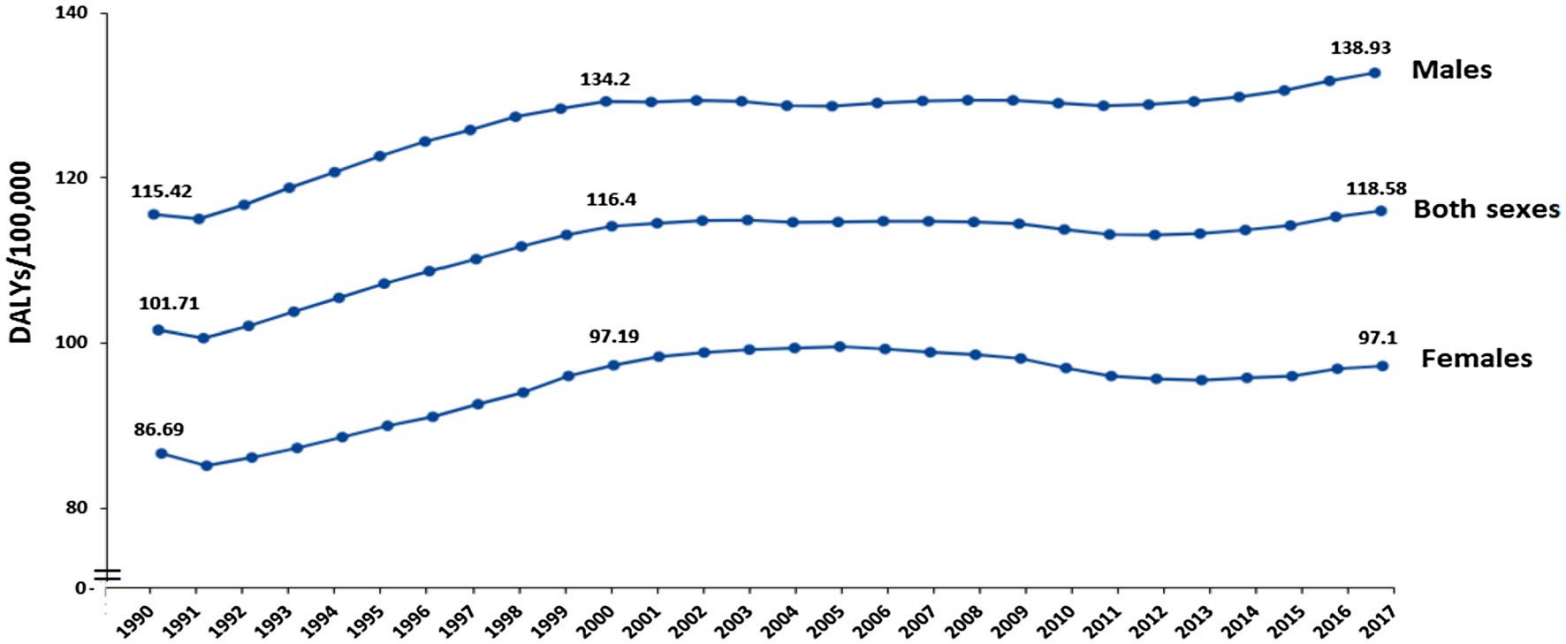
Disease burden of substance use disorders (disability-adjusted life years per 100,000) in Somalia ( Source: Global Burden of Disease [38])

There is scant information available on the prevalence of substance abuse in Somalia [39]. Figure 4 presents the disease burden of substance use disorders in Somalia, mainly khat but also opioid, cocaine, amphetamine, and cannabis. The trend shows that from 1991 (onset of the civil conflicts) substance use increased in both sexes until 2000 and then almost plateaued. However, in recent years a tendency of increased drug use can be seen. Nevertheless, the disease burden of substance use disorders has the lowest values in Somalia among the EMR countries. To date, the most commonly used substance is khat, a psychoactive substance indigenous to East Africa and the Arabian Peninsula [39–41]. Although in the past chewing khat was a traditional social norm commonly consumed primarily by adult men, the consumption and patterns of use has shifted and now increased addiction is being seen in women and youth [39–41].

#### Physical/mental symptoms and collective trauma

A study on Somali refugees showed that those exposed to collective or personal trauma experienced more psychological distress [42]. Several studies show significant mental health issues associated with conflict and refugee settings [3, 24, 42, 43]. The same is true for Somalis within the country and as refugees [3, 24, 43, 44]. These psychological experiences have been diagnostically classified under the categories of PTSD, anxiety and depression. Somalis also acknowledge feelings of hopelessness, despair, anxiety and anguish as part of their symptomatology, despite not explicitly labelling such experiences and symptoms as PTSD or depression [42, 43]. The consequences of trauma among Somalis have also been described in somatic terms with emphasis on headaches and other unexplained body pains that seem untreatable with physical health remedies offered by health services [24, 43, 44].

### Social and cultural determinants

Somalis’ perceptions of psychological wellbeing are dichotomised like health and illness, that is, there are only two categories: mentally well and mentally ill people. Mild forms of mental disorders are usually neglected and not considered as serious problems requiring any psychological assistance [24, 35]. Since cultural and faith healing are deeply rooted and widely practiced by Somalis [43, 44], understanding of the causes of mental illness or psychosocial distress are often seen as spirit possession (*jinn*), witchcraft (*sihr*), or evil eye (*eel*). Treatments include exorcism (*ruqya*) and the recitation of verses of the Qur’an by the individual, family members, or imams and other religious or traditional healers [43, 44]. The belief in cultural and spiritual causality of psychosocial illness has a profound effect on the understanding of illness and help-seeking for people with such beliefs. In fact, across the country, cultural and spiritual aspects remain the most important attribute of psychosocial illness and, as such, first line treatments typically involve some form of religious and traditional healing [45].

Significant stigma and discrimination in Somali society conceals mental health issues [3, 24]. Stigma has damaging effects like distancing from a family diagnosed with mental illness and reluctance to seek clinical help or professional assistance. Stigma is, therefore, one of the main barriers to engaging with mental health services in Somali society [3, 24]. Health service seeking is also hindered by cultural gender norms in the society. Normally, women refrain from discussing rape and sexual assault with care providers, or to seek mental health treatment from medical professionals. Men, on the other hand, often choose self-medication in the form of substance use, mostly khat [3, 4].

Due to the collapse of the nation state and community reliance on spiritual healing, there has been an explosion of spiritual healing programmes popular known as *ilaaj* (healing in Arabic). Although these centres are widespread and highly utilised, currently limited information is known about them in terms of the numbers, evidence supporting their treatment and the number of clients in their care. While crucial as community resources, there is documented evidence of human rights transgressions in the form of chaining and exorcism-related physical abuse [13, 46].

### Discussions and recommendations for priority setting in mental health in conflict settings

As the country staggers toward political, social and economic recovery from the nearly four decades of conflict and lack of strong central government, it is crucial to lay a solid foundation of sound mental health policies, strategies and infrastructures to address the myriad needs of its citizens. In this paper, we propose the following four key strategies to address the monumental mental health needs of the country. These are: establishing and strengthening governance for mental health and effective leadership, implementing human resource policies, regulation and training, provision of comprehensive integrated community-based mental health services through PHC and mental health financing.

#### Establish and strengthen governance for mental health and effective leadership

Strong and effective mental health governance overseen at a senior ministerial level is needed to coordinate activities of the department of mental health at federal and state levels. Such a high-level portfolio is crucial to address the development and maintenance of an integrated mental health system as part of the general health care system. Mental health leadership is required to develop, regularly review and update policies, strategies and legislation [47, 48]. There is a need for a mental health department/directorate responsible for development, coordination, monitoring and evaluation that is adequately staffed and resourced. Chief among health infrastructure needs is to create an inclusive mental health policy and strategy that is backed by a progressive mental health legislation [49]. Considering the systemic neglect and abuses of those with mental illness at community and health facilities, it is prudent to have a mental health law that is clearly aligned with the United Nation’s Convention of Rights of Persons with Disabilities (CRPD) human rights framework [50]. The Federal Republic of Somalia has already ratified and is signatory to the CRPD and as such, any mental health law must follow the letter and spirit of the CRPD to ensure the rights of persons with mental illness and other psychosocial disabilities are fully protected under the law of the land and specifically laws pertaining to their treatment in the community and service systems.

#### Implement human resource policies, regulation and training

Secondly, the country needs robust health education, certification and training policies, and strategies with legal backing. Currently Somalia faces a chronic shortage of health workers which is a significant barrier in achieving UHC. In addition, due to years of an unregulated market, the country faces the risk of an unregulated and unlicensed body of health professions whose qualifications and competencies cannot be ascertained. It is within this context that a national regulatory authority is urgently required to take stock of health service providers, health learning institutions and health practitioners. Then the country needs to develop ways to assess the quality of services, education and qualifications.

One of the key innovations required is to develop an appropriately skilled workforce who can work in community setting adopting a multidisciplinary teamwork ethos, integrated into PHC, linked with expertise for referral and inpatient care, networked with local resources in a collaborative way to deliver efficient and cost- effective mental health care. This requires a transformation of roles and responsibilities of general health workers and specialist mental health service providers, such as through various types of task-sharing, and developing new cadre for mental health professionals like community mental health workers and case managers. There is a need to build the capacity of non-specialist health workforce and specialist mental health workforce to provide community-based integrated care for priority mental disorders [10].

#### Provide comprehensive and integrated mental health in community-based settings through a PHC approach

The UN adopted a resolution to support UHC in 2012 and urged countries around the world to provide quality comprehensive health services through PHC. In line with UHC and PHC, WHO developed the Mental Health Action Gap (mhGAP) guide to scale up treatments in resource limited regions. The mhGAP model is likely the most appropriate model for the Somali context as the model emphasises a non-specialty approach to service provisions where mental health support and treatment can be provided by non-specialist health workers (nurses, midwives, GPs) and community health workers at PHC and community entry points [51].

The mhGAP guide is a guideline to address the growing burden of mental, neurological and substance use disorders, extremely low number of mental health professionals, and shortage of mental health facilities in many LMIC [52]. The guide identifies priority conditions based on a high disease burden (in terms of mortality, morbidity and disability), large economic costs, and human rights violations. The priority conditions include depression, psychoses, trauma-related conditions (including PTSD), epilepsy, child and adolescent mental and behavioural disorders, dementia, disorders due to substance use, and suicide/self-harm. The aim of mhGAP is capacity building of LMIC in managing mental, neurological and substance use disorders by training non-specialists to detect and manage these illnesses. The guide also facilitates delivery of the mhGAP evidence-based guidelines in non-specialised health care settings. The mhGAP has been successfully put into context in several LMIC settings [53–55]. The mhGAP has been introduced in two universities of Somaliland: The University of Hargeisa and Amoud University, with the intention of further cascading across the semi-autonomous region [56].

#### Mental health financing

To achieve UHC, a key target of the Sustainable Development Goals [57], countries need sufficient quality of health services, financial coverage or risk protection, ensuring that the services do not expose the user to financial hardship [58, 59]. Discerning with this lens, mental health issues are neglected globally [60]. In resource poor countries, people with mental disorders have negligible access to quality health care and are consequently vulnerable to suffering and ill health [61]. Mental health patients in such situations suffer from human rights abuses, poverty, stigma, discrimination and even premature death [28, 61, 62]. WHO Special Initiative for Mental Health (2019–2023) specifically focuses on universal coverage for mental health. WHO stresses that there is no health or sustainable development without scaling up and integrating mental health services to PHC. Somalia has the opportunity to closely collaborate with WHO and international partners to ensure mental health is a priority condition and its financing prioritised through substantial national budgetary allocation as well as external developmental support.

### Limitations of the study methodology

The search terms used for the systematic mapping were applied to capture a wide range of mental health studies addressing Somalia. However, some specialised embedded niches of research, e.g., childhood mental health and mental health effected social development might be underrepresented. Moreover, lack of publications originating from Somalia and traditions regarding the dissemination of findings in foreign journals may misrepresent most of the indigenous mental health issues in the searched results. In addition, whilst a number of recommendations are made, further research is warranted to assess the effectiveness of the proposed interventions in Somalia.

### Conclusion

This paper outlines the key issues of mental health in Somalia that require researchers, clinicians, administrators, programme planners and policy makers to comprehend the magnitude of the factors that moderate them. The vast scale of the mental health problems in Somalia elucidated in this paper warrant a dire need that the Somali government and its national/international partners should prioritise and emphasise investments in the prevention and the treatment of mental illness across the country. Based on available data/information and evidence-derived in this paper, guidelines are provided to policy makers in setting priorities for the designing of the mental health system and delivery of interventions to promote mental health and psychosocial wellbeing in Somalia.

## Data Availability

The data that support the findings of this study are openly available in the WHO, Global Health Observatory. Geneva: World Health Organization. https://www.who.int/data/gho.

## Abbreviations

ACE: Adverse Childhood Experience
CRPD: Convention of Rights of Persons with Disabilities
DALY: Daily Adjusted Live Years
EMRO: Eastern Mediterranean Regional Office
GRT: Gruppo per le Relazione Transculturali
HRH: Human resources for health
HRW: Human Rights Watch
IOM: International Organization for Migration
JMCNA: Joint Multi-Cluster Needs Assessment
LMIC: Low to Middle Income Countries
MHPSS: Mental Health and Psychosocial Support
MNS: Mental neurological and substance use disorders
NGO: Non-Governmental Organization
PHC: Primary Health Care
PTSD: Post-traumatic stress disorder
UHC: Universal Health Coverage
UN: United Nations
WB: World Bank
WHO: World Health Organization
